# Operative treatment of cervical radiculopathy: anterior cervical decompression and fusion compared with posterior foraminotomy: study protocol for a randomized controlled trial

**DOI:** 10.1186/s13063-021-05492-2

**Published:** 2021-09-08

**Authors:** Marek Holy, Anna MacDowall, Freyr Gauti Sigmundsson, Class Olerud

**Affiliations:** 1 Department of Orthopedic Surgery, Örebro University School of Medical Sciences, Örebro University Hospital, Örebro, Sweden; 2 Department of Surgical Sciences, Uppsala University Hospital, Uppsala, Sweden

**Keywords:** Anterior cervical decompression, Anterior cervical discectomy, Posterior foraminotomy, Randomized control trial

## Abstract

**Background:**

Cervical radiculopathy is the most common disease in the cervical spine, affecting patients around 50–55 year of age. An operative treatment is common clinical praxis when non-operative treatment fails. The controversy is in the choice of operative treatment, conducting either anterior cervical decompression and fusion or posterior foraminotomy. The study objective is to evaluate short- and long-term outcome of anterior cervical decompression and fusion (ACDF) and posterior foraminotomy (PF)

**Methods:**

A multicenter prospective randomized controlled trial with 1:1 randomization, ACDF vs. PF including 110 patients. The primary aim is to evaluate if PF is non-inferior to ACDF using a non-inferiority design with ACDF as “active control.” The neck disability index (NDI) is the primary outcome measure, and duration of follow-up is 2 years.

**Discussion:**

Due to absence of high level of evidence, the authors believe that a RCT will improve the evidence for using the different surgical treatments for cervical radiculopathy and strengthen current surgical treatment recommendation.

**Trial registration:**

ClinicalTrials.gov NCT04177849. Registered on November 26, 2019

## Administrative information

Note: the numbers in curly brackets in this protocol refer to SPIRIT checklist item numbers. The order of the items has been modified to group similar items (see http://www.equator-network.org/reporting-guidelines/spirit-2013-statement-defining-standard-protocol-items-for-clinical-trials/).

**Table:** 

Title {1}	Operative Treatment of Cervical Radiculopathy: Anterior Cervical Decompression and Fusion Compared with Posterior Foraminotomy: Study protocol for a randomized controlled trial
Trial registration {2a and 2b}.	ClinicalTrials.gov Identifier: NCT04177849.
Protocol version {3}	V1, 2019-12-19
Funding {4}	Institutional and CSRS Grant
Author details {5a}	1) Marek Holy MD. Department of Orthopedic Surgery, Orebro University School of Medical Sciences, Orebro University Hospital, Orebro, Sweden 2) Anna MacDowall MD. PhD. Department of Surgical Sciences, Uppsala University Hospital, Uppsala, Sweden. 3) Freyr Gauti Sigmundsson MD. PhD Department of Orthopedic Surgery, Orebro University School of Medical Sciences, Orebro University Hospital, Orebro, Sweden 4) Claes Olerud Prof. MD. PhD Department of Surgical Sciences, Uppsala University Hospital, Uppsala, Sweden.
Name and contact information for the trial sponsor {5b}	No sponsor.
Role of sponsor {5c}	The funder played no part in study design; collection, management, analysis, and interpretation of data; writing of the report; and the decision to submit the report for publication.

## Introduction

### Background and rationale {6a}

Cervical radiculopathy is a symptom complex consisting of arm pain, impaired sensory and motor function in the corresponding dermatomes and myotomes, and varying degrees of neck pain. The cause is often foraminal stenosis, which is secondary to degenerative disk disease with disk herniation (21.9%) and/or osteophyte formation from the uncovertebral or facet joints (68.4%) compressing the nerve in the root canal [1, 2]. The incidence has been estimated at 83.2 per 100,000 inhabitants/year with a peak at 50–54 years. Men are affected more often than women (107.3 vs. 63.5 per 100,000/year). The most often afflicted nerve root is the C7 root (46.3%), followed by C6 root (17.6%) [2].

The diagnosis is set by the typical history and findings, which often involves loss of sensory and motor function, and diminished deep tendon reflexes. The foraminal compression test is a provocation test for the affected nerve root. The head is extended and rotated towards the affected side. A positive response is when pain is reproduced by axial compression of the head. The clinical results must correlate with the findings of the neuroradiological examination, primarily the MRI but alternatively CT is used in cases where MRI cannot be performed, because of, claustrophobia, non-MRI-compatible pacemaker or dorsal column stimulator, or the presence of other metal objects that may cause damage tissue by shifting position under the exam [3]. In ambiguous cases, neurophysiological examinations may also be valuable.

Spontaneous restitution is common; hence, non-surgical treatment is often the first choice. Non-operative treatment may consist of pain medications, a neck collar, and physiotherapy. Indications for surgery are failure off non-operative therapy, with no relieve if the pain after a period of a couple of months, or if complications occur, i.e., intractable pain, progressive paresis, or cervical myelopathy. In these cases, surgical intervention may result in a reliable improvement and enhanced quality of life [4].

Theoretical advantages of anterior decompression and fusion (ACDF) are the direct removal of the pain-generating disk fragment or osteophyte compression applied to the nerve root. Drawbacks are the approach-related complications, such as injury to neurovascular or other structures, and pseudoarthrosis, which may occur in a number of cases. The most feared acute complication is a postoperative hematoma, which, if untreated, may rapidly lead to airway obstruction or compression of the spinal cord. This occurs with an incidence of 1%. The incidences of other known complications with ACDF are as follows: esophagus lesion 0.25% [5], infection 0.1–1.6% [6], injury to the recurrent laryngeal nerve 0.6–2.9% [7], injury to the superior laryngeal nerve 0–1.25% [8], injury to the hypoglossal nerve 0–1.28% [9], vertebral artery injury 0.08% [10], dural tear 0.5–3.7% [11], spinal cord injury 0–0.24% [12], Horner’s syndrome 0.06% [13], brachial plexus injury 0.1% [14], C5 palsy 0–2.5% [15], and injury to the thoracic duct 0.08% [16].

Other complications associated with the anterior approach include intermittent early dysphagia which in most cases resolves [17]. In addition, adjacent segment disease (ASD) may occur, as spinal fusion has been blamed for increasing the incidence of degeneration in adjacent levels [18]. 25.6% will develop ASD within 10 years after ACDF and 7.5% will need further surgery. However, the cause for ASD is controversial, as disk degeneration is an age-related process affecting all disks and ASD may be a normal progression of the degenerative process affecting the disk adjacent to the fusion [19]. ACDF leads to clinical success in 83–91% of cases, with a reoperation incidence of 4–14% [20, 21].

Theoretical advantages with posterior foraminotomy (PF) include the following: fewer vital structures can be injured during the primary procedure and the segment is left unfused perhaps decreasing the risk for ASD. A disadvantage is that the decompression is indirect, meaning that the compressing fragment or the osteophyte is not removed, but the nerve root is allowed to move away from it, as the “roof” of the foramen is opened.

Approach-related complications with PF are postoperative hematoma, which may compress the spinal cord, and C5 palsy [15], where the exact pathophysiology is not fully understood.

Instability issues after partial facetectomy during PF may lead problems that require fusion, in general fusion after PF have rates of up to 5%. Recent retrospective studies of minimal invasive PF with over 1000 cases shown that a good level of decompression is achieved, i.e., to same or better NDI in comparison to ACDF [22–32].

The incidence of ASD is 6.7% 10 years after one level of PF, with 3.2% requiring reoperation for ASD [33]. The preserved motion may lead to restenosis as the degeneration continues with the risk of secondary surgery on the index level [34]. PF will lead to clinical success in 64–96% with a reoperation incidence of 4–7% in retrospective cohort studies [22–32, 35–37].

Both methods result in a high rate of clinical success with a low incidence of reoperations. However, there are no prospective controlled studies with a high level of evidence comparing the two approaches. High level of evidence from RCTs could improve treatment guidelines and recommendations for the surgical treatment off cervical radiculopathy [38–41].

### Objectives {7}

The main objective of the trial is to compare the clinical outcome of ACDF surgery after 2 years of follow-up with the outcome of PF in patients with cervical radiculopathy by using patient-reported outcome measures (PROMS). The primary outcome variable is the Neck Disability Index (NDI) at the 2-year follow-up [42]. Secondary outcome variables are the European Quality of Life-5 Dimensions (EQ-5D) [43, 44] and the Numeric Rating Scales (NRS) [45] for arm and neck pain. To evaluate temporal differences between the recoveries after each surgical technique, the variables will also be collected after 4 weeks, 3 months, and 1 year. Tertiary variables are complications and secondary surgeries.

### Trial design {8}

The trial is conducted as a multicenter clinical randomized controlled trial (RCT) with 2 years of follow-up using PROMs and radiological parameters. The RCT will be carried out according to the SPIRIT and CONSORT statements for clinical trial reporting [46, 47]. Randomization without stratification in a 1:1 ratio using the SMART-TRIAL software (www.smart-trial.co) will result in allocation to either ACDF or PF, a 2-arm parallel group design. The primary aim is to evaluate if PF is non-inferior to ACDF using a non-inferiority design with ACDF as “active control.” A secondary superiority design will also be applied. In the non-inferiority design, the hypothesis is that PF is equal to ACDF. The difference between PF and ACDF is not more than the MCID for NDI, i.e., not more than 7.5 points or 15% units [48–50].

## Methods: participants, interventions and outcomes

### Study setting {9}

Participating centers are Örebro University Hospital (primary site), Uppsala University Hospital, Umeå University Hospital, and Ryggkirurgiskt Centrum Stockholm. Each center has high volume of cervical radiculopathy patients and complex spine surgery teams to provide the interventions in form of PF and ACDF

### Eligibility criteria {10}

#### Inclusion

The inclusion criteria are as follows: cervical radiculopathy; age 18–65 years; patients with symptoms of radiating arm pain with duration of at least 6 weeks; neck disability index (NDI) over 30 points (60%); correlating findings on MRI, one or two consecutive cervical levels; eligible for both treatments; and ability to understand and read Swedish language.

#### Exclusion

The exclusion criteria are as follows: previous cervical spine surgery; more than two cervical levels requiring treatment; severe facet joint osteoarthritis; symptoms or marked radiologic signs of myelopathy; drug abuse, dementia, or otherwise expected low compliance; cervical deformity or marked instability (3.5-mm translation or > 11 degrees more motion at index level compared to adjacent segments [51]; history of severe cervical trauma, WAD or generalized pain syndrome; pregnancy; rheumatoid arthritis or ankylosing spondylitis; malignancy; active infection or another severe systemic disease; or when the surgeon deems the participant unsuited for either of the interventions.

### Who will take informed consent? {26a}

The consultant spine surgeon will take informed consent during consultation meeting with the patient that is eligible for inclusion.

### Additional consent provisions for collection and use of participant data and biological specimens {26b}

Eligible patients will be informed in the clinic about the study and included if consent is given. Study information is given by the consultant spine surgeon. No other consent provisions are applicable; no biological specimens are collated.

### Interventions

#### Explanation for the choice of comparators {6b}

Treatment for CR in Europe and USA has been ACDF and is considered “Golden standard,” yet in the Asian Pacific, the main treatment for CR is PF. No level one evidence has been published yet; the investigators believe that clinical equipoise is between ACDF and PF.

### Intervention description {11a}

#### Anterior cervical decompression and fusion

A 4-cm anterolateral transverse incision is made over the index level, on either the right or left side according to the surgeon’s preference. Platysma is sectioned transversal to its fibers, and the anterior aspect of the spinal column is bluntly exposed between the carotid sheath and the esophagus. The disk is excised, including the posterior longitudinal ligament. Disk fragments and/or osteophytes from the uncovertebral joint on the affected side are removed until the root is fully decompressed. Reconstruction is typically done with an interbody spacer, autologous bone graft, and a stabilizing plate screwed to the adjacent vertebral bodies. A fusion cage with integrated screws may also be used according to the surgeon’s preference [20, 41].

#### Posterior foraminotomy

A 4-cm longitudinal midline incision exposes the spinous processes of the adjacent vertebrae. The facet joint covering the index foramen is exposed through intermuscular planes. The root canal is opened as the medial third of the facet joint is removed. The affected nerve root is decompressed by laterally undercutting the facet joint throughout its length [36].

#### Criteria for discontinuing or modifying allocated interventions {11b}

If severe adverse events are noted, the attending spine surgeon will contact the investigator of the study. Analysis of severe adverse event will be conducted to evaluate if the method is harmful and then decide to stop the trial. Stopping criteria is difference of over 30% in NDI between the outcomes, i.e., to more than 2x MCID on any of the follow-up PROMS at 4–6 weeks, 3 months, 1 year, and 2 years. Participants can at any time request to drop out of the study.

#### Strategies to improve adherence to interventions {11c}

Study supervisor will monitor PROMS and radiological parameters; if the participant fails to attend, a written remainder for PROMs, CT scan, and MRI will be sent by mail. This is done on two occasions and with the last and third reminder which is with a phone call. Overall, 3 reminders will be given to the participant before exclusion for non-adherence is conducted.

#### Relevant concomitant care permitted or prohibited during the trial {11d}

Postoperative care, pain control, and physiotherapy will be identical in both groups. The physiotherapy will aim at informing the patient about general mobilization. Neck training will consist of muscular control in the initial phases with range-of-motion exercises starting from 6 weeks postoperatively. Stiff neck collar that affects range of motion is prohibited.

#### Provisions for post-trial care {30}

Patients have in general a standard insurance in case of malpractice and harm provided by the Swedish healthcare system. After the trial has been conducted, the study patients are still eligible for treatment for cervical disorders if needed.

### Outcomes {12}

#### Primary variable

The primary outcome, NDI, is a self-administered questionnaire with 10 items measuring disability in patients with neck pain. The questions cover daily activities, such as the ability to dress, lift heavy objects, read, work, drive a car, sleep, and perform leisure time activities, as well as the amount of pain, headache, and concentration abilities. Each item is scored from 0 to 5. The disability is more severe with higher scores, while the maximum score is 50 points. The score is transformed into a percentage score (range 0 to 100%). The minimum clinically important difference (MCID) is considered to be 7.5–8.5 [48, 49] or 17.3% [50]. NDI PROMS are collected pre operation and after 4–6 weeks, 3 months, 1 year, and 2 years.

#### Secondary variables

Secondary variables are the European Quality of Life-5 Dimensions (EQ-5D; with a range from approximately − 0.5 to 1, with higher scores indicating better quality of life) using the Swedish translation [43, 44].

Numeric rating scales (NRS) are used to assess arm and neck pain. The scales range from 0 to 10, 0 being the best and 10 the worst. MCID for NRS neck and arm is not yet determined [45].

#### Tertiary variables

Tertiary variables, complications, and reoperations will be collected as they may occur.

#### Radiology

The following radiological examinations will be performed: X-ray radiograph in flexion/extension of cervical spine, and MRI and CT scan will be carried out preoperatively and after 1 and 2 years. In order to evaluate the degree of decompression, a postoperative CT will also be performed. The degree of restenosis over time, development of adjacent segment pathology, facet joint degeneration, range of motion changes, and fusion healing will be evaluated.

### Participant timeline {13}

Patient are randomized and enrolled in the study at the consultation with spine surgeon; there is usually 4–18 weeks waiting before surgery. During this time, BASELINE data is collected. After intervention, CT scan is performed then the patients are followed with PROMS and radiological studies after 4–6 weeks, 3 months, 1 year, and 2 years.

**Table:** 

OMSAP	Study period					
	Enrolment	Surgery	Post-allocation			
**Timepoint**	***− 4–18 weeks***	**0**	***4–6 weeks***	***3 months***	***1 year***	***2 years***
**Enrolment:**						
**Eligibility screen**	X					
**Informed consent**	X					
**Allocation**	X					
**Interventions:**						
***[ACDF OR PF]***		X				
**Assessments:**						
***[MRI, CT, Flex/Ext-X-ray]***	X				X	X
***[NDI, NRS, EQ-5D]***	X		X	X	X	X
***[Post Op. CT]***		X				

### Sample size {14}

The MCID in the NDI is approximately 15% [50], and the standard deviation (SD) in the NDI is 25%. With these parameters, a two-sided superiority trial with significance level at 0.05 and power at 80% will require 44 patients in each group. To make up for crossovers, noncompliance, and follow-up losses, the total number of patients is set at 110.

### Recruitment {15}

Participating centers, Örebro University Hospital (primary site), Uppsala University Hospital, Umeå University Hospital, and Ryggkirurgiskt Centrum Stockholm have been chosen for their high volume of cervical cases with cervical radiculopathy. These patients are generally referred by a general practitioner and are eligible for consultation with a consultant spine surgeon. To achieve a fast and easy enrollment, a web-based software, Smart-Trials, is used to register consent, inclusion, and randomization. A written consent is also given during the consultation with spine surgeon.

## Assignment of interventions: allocation

### Sequence generation {16a}

Randomization is achieved by registering the patient in Smart-Trials software (www.smart-trial.com), which will generate allocation to either ACDF or PF. It is not possible to change group after randomization.

### Concealment mechanism {16b}

Concealment mechanism is provided by the software, which allows the user to preform randomization without the possibility of knowing the outcome beforehand.

### Implementation {16c}

Each spine surgeon has individual logging, and the log-on process is with a two-stage verification using logging and mobile phone SMS code. The spine surgeon implements the allocation.

## Assignment of interventions: blinding

### Who will be blinded {17a}

Blinding is not possible; the interventions are different and neither the patient nor the surgeon are blinded. X-ray assessment is not possible to blind.

### Procedure for unblinding if needed {17b}

No blinding

### Data collection and management

#### Plans for assessment and collection of outcomes {18a}

The Swedish Spine Register [52] is used for gathering data. Patient-reported outcome measures (PROMs; NDI and EQ5-D [48–50]) are sent to the patient by mail at 1 and 2 years of follow-up. PROMs for the 4–6 weeks and 3 months follow-up are not standard to the SweSpine protocol which is why these are mailed separately to the patients. The MRI, CT, and flexion-and-extension radiographs are assessed by 2 spine surgeons independently. In case of non-consensus, a discussion will be performed until consensus is reached. Standard protocols for MRI cervical spine and CT cervical spine are used.

#### Plans to promote participant retention and complete follow-up {18b}

Patients that do not reply on the first set of PROMs will get two reminders by mail. If still no response, they will be approached once by telephone, after which they will be excluded. Patients that choose to opt out will be excluded upon their request. Outcome PROMs and radiology will be collected for the time they remained in the study, unless the patient demands that this data is removed.

### Data management {19}

The primary site is where all the study data will be stored in a protected database; sources of collected data are radiology outcomes and PROMS. Radiology outcomes are collected from all the sites and stored digitally on secure drive with backup provided by the Department of Radiology, secured by 1-stage verification login.

PROMS outcome are collected by two different pathways: first is by SweSpine for 1- and 2-year data and by the primary site for 4–6 weeks and 3 months data. SweSpine data are accessible by one-stage verification login on the SweSpine main page. SweSpine data can be viewed by the spine surgeons with the patient’s ID. This data can only be accessed for study purpose and with Swedish Ethical Review Authority’s approval. Upon retrieval of 1 and 2 years data by the investigator, this is then stored on a hard drive with backup and 1-stage verification login.

PROMS for 4–6 weeks and 3 months are manually collected and converted to digital form by the primary site by the controller and investigator. All PROMS data are checked by the controller and investigator before analysis. All PROM data will be processed by using IBM SPSS statistics or other equivalents by the investigator with support of a statistician; also, this data will be password protected.

Data from Smart-Trials is not used as outcome measures and is stored upon completion in a database accessible only by the primary investigator.

### Confidentiality {27}

All data management and analysis are done using unidentified PROMS data; only the investigator team has access to the ID of the recruited participants. Trial data will not be shared with other researchers.

### Plans for collection, laboratory evaluation, and storage of biological specimens for genetic or molecular analysis in this trial/future use {33}

No laboratory test or biological materials are collected during this trial.

## Statistical methods

### Statistical methods for primary and secondary outcomes {20a}

Primarily, patient-related outcome measures will be analyzed in terms of intention to treat (ITT) and include all randomized patients. Using analysis of covariance (ANCOVA), the mean outcome values for each treatment group will be analyzed, with adjustments for baseline values. The mean difference between the groups will be presented.

Secondary outcome analyses using the Student *t* test, chi-square, Mann-Whitney, and Fisher exact test.

The tertiary outcome analyses will be based on available cases. The time to revision surgery according to treatment assignment will be analyzed and plotted according to the Kaplan-Meier method, while hazard ratios, regarding secondary surgery after ACDF compared with PF, will be estimated by the Cox model with calendar time as the time scale. Men and women will be analyzed separately.

### Interim analyses {21b}

An interim analysis will be performed by an independent observer when 40 patients are included, regarding NDI difference and adverse effect [53]. If severe adverse events are noted, the attending spine surgeon will contact the investigator of the study.

### Methods for additional analyses (e.g., subgroup analyses) {20b}

No primary subgroup analyses are planned.

### Methods in analysis to handle protocol non-adherence and any statistical methods to handle missing data {20c}

Missing data will be imputed using multiple imputation [54].

### Plans to give access to the full protocol, participant level-data, and statistical code {31c}

No later than 3 years after the collection of the 2-year data after the randomization PROMS, we will deliver a completely deidentified dataset if requested.

## Oversight and monitoring

### Composition of the coordinating center and trial steering committee {5d}

The coordinating center is Örebro University Hospital and the steering committee is as follows:

Study director: Prof. Claes Olerud MD. PhD has overall supervision of study.

Principal investigator: Marek Holy MD. Responsible for recruitment, data collection, adherence to study protocol

Main coordinator and controller: Hanna Wennerlund, responsible for PROMS, Radiology

Critical reviewer: Anna MacDowall MD. PhD, Freyr Gauti Sigmundsson MD. PhD

Independent observer: Associate Prof. Acke Ohlin MD. PhD. Responsible for interim analysis and auditing trial conduct.

The local centers have each a coordinator that is in direct contact with main coordinator and controller. This is to secure date of inclusion, scheduling time of intervention, and monitoring radiology outcomes and baseline.

Recruits are referred to each center by their general practitioner or family doctor. The senior spine surgeons at the main and local centers are individually responsible for recruitment and inclusion to study and randomization and also to provide the surgical intervention; this is facilitated by the Smart-Trials software.

### Composition of the data monitoring committee, its role and reporting structure {21a}

Data monitoring is performed on a weekly basis by the main coordinator and controller with support of the local coordinator; this is to ensure that all the events, i.e., PROMS, radiology, is performed according to protocol. Data monitoring committee comprises of:

Principal investigator: Marek Holy MD. Responsible for recruitment, data collection, adherence to study protocol

Main coordinator and controller: Hanna Wennerlund, responsible for PROMS, radiology

Interim analysis is also performed after the 40 participants have been included and surgical intervention is performed, by the independent observer.

Independent observer: Associate Prof. Acke Ohlin MD. PhD

### Adverse event reporting and harms {22}

Adverse events (AE) that are anticipated are infection, implant failure, neurologic impairment post-surgery, and other rare approach-related complications like esophagus perforation and nerve injury. AE are reported by the senior spine surgeon that performed the surgical intervention to the primary investigator.

Serious adverse events (SAE) are not anticipated; they are reported by the senior spine surgeon according to the Swedish healthcare standards which depend on the type of SAE. Such SAE may include deep vein thrombosis with pulmonary embolism and subsequent death, death due to infection, and meningitis or similar infections.

### Frequency and plans for auditing trial conduct {23}

The independent observer performs audits conducted across all sites when 40 participants are included, based on the 4–6 weeks, 3 months, 1 year, and 2 years follow-up data. In addition, analysis of any adverse events will be performed by the independent observer.

### Plans for communicating important protocol amendments to relevant parties (e.g., trial participants, ethical committees) {25}

Any change in the protocol from the primary protocol that is approved by the Swedish Ethical Review Authority must be reported and resubmitted for approval, that is, equal to changes in intervention, outcome measures, and sample size.

### Dissemination plans {31a}

The trial result will be submitted to peer review journals and presented at international meetings.

## Discussion

ACDF and PF are two common techniques for treating cervical radiculopathy. Both methods seem to result in a high frequency of clinical success with low incidences of complications and reoperations according to retrospective data, but at different costs as no implants are utilized in PF [37]. In an observational registry study, we did not find any difference between the two methods in terms of clinical outcome, complications, and reoperations [34]. As there may be undetected selection bias and underreporting of complications in a registry study, the results need to be confirmed in studies with higher level of evidence.

In most instances, a root canal stenosis is caused by osteophyte formation from the uncinate process and/or disk protrusion compressing the root from the anterior. The most determining factor for clinical success seems to be how well the nerve root is decompressed. A recent systematic review and meta-analysis of minimally invasive PF showed that arm pain improvement was greater in the PF group compared to the ACDF group, thus supporting the hypothesis of sufficient decompression with PF [55]. In ACDF, the compressing elements can be removed directly, whereas in PF, the decompression is indirect; by removing the “roof” of the root canal, the nerve root is allowed to move away from the compressing elements. Thus, the mode of decompression is fundamentally different between the two methods.

The level of decompression that is shown in retrospective series seems to be sufficient for both methods, i.e., to the range of 90% or more. However, PF shows larger variation in clinical success with range of 64–96% [35–37] and ACDF 83–91% [20, 21]; the reason for this variation is not exactly clear.

The issue with restenosis at the index level of surgery and the need for re-decompression of the same nerve-root is another issue that needs more elaborate study. We know from prior studies that non-specified reoperation rates are in the range of 4–7% in PF and 4–14% in ACDF. We think that these data are not comparable and that the reasons for reoperation on index level are different. For PF, it will be the issues of inadequate decompression, but for ACDF, it will be inadequate decompression, hardware failure, and/or fusion problems, e.g., loosening or pseudoarthrosis. We believe that PF may in the long term have unremitting degeneration issues and that the risk for restenosis will increase over time. The reason may be the partial facetectomy in a loadbearing joint, in which the degeneration will continue [34].

The main question of this study is to evaluate if there is equal outcome between the two methods. Other questions are if complications and reoperations differ. We chose NDI as the primary outcome measure. The instrument has been used in multiple previous studies on cervical radiculopathy and focus on the pain and dysfunction associated with radiculopathy, i.e., is disease specific and allows comparison to previous work. We also include VAS separately for arm and neck pain as we believe this possibly separates the success of decompression from the morbidity associated with the different approaches. The EQ-5D was included as this is a non-disease specific quality-of-life instrument allowing comparison to other ailments.

The cost-risk benefit for ACDF vs. PF needs to be considered; the approach-related complications and reoperations rates are important from financial perspective. ACDF have more implications than PF due to the anatomical facts of an anterior approach; this is in favor for PF. Implants are not utilized for PF; this lowers the coast of surgery in favor for PF. Surgical selection bias is a factor that must be considered when looking at retrospective studies of ACDF vs. PF. The difference in various clinical settings may influence the choice of method as factors such as multilevel vs. single level surgery, smoking vs. non-smoking, and prior instability on dynamic radiographs. The most decisive difference between ACDF and PF is the fusion at the index level. In previous studies on cervical radiculopathy, motion preservation using artificial disks seem to decrease the incidence of radiological adjacent segment pathology (RASP) next to a fused segment. But when it comes to clinically relevant adjacent level pathology (CASP), especially if leading to surgery, the data is less compelling with a 7.1–8.5% reoperations rate in ACDF for CASP [56, 57]. Our study design, with X-ray, MRI, and CT at regular intervals, will allow us to shed some additional light on the unsettled issue if lost or maintained motion at the index segment and will protect from CASP in the long-term.

## Limitation

General limitation of RCT is patient selection and external validity of the trial. The generalizability of this study may be limited by the exclusion of patients with concomitant cervical diseases in addition to CR and the level of degeneration; this is equal to the age selection of 18–65 years. Selections bias is also factor that is present due to the exclusion criteria; another limitation is that the surgeons may not be equally skilled at performing ACDF and PF. A surgeon must be proficient in both PF and ACDF techniques; the minimum requirement is 10 surgeries. The use of NDI for CR is not how NDI was intended; NDI is an instrument for whiplash patients but is used in general for CR studies [42]. The use of EQ-5D is also debatable; EQ-5D is a non-specific general health questionnaire. Follow-up is a limiting factor; 2-year follow-up is good to see how good decompression and pseudoarthrosis and hardware failure rates are. But for ASD and restenosis on index level, a longer FU is needed.

## Trial status

Protocol version Nr: V1, 2019-12-19, recruiting from January 20, 2020, approximate date of recruitment completions January 1, 2023

Swedish Ethical Review Authority Number: Dnr 2019-00003

ClinicalTrials.gov Identifier: NCT04177849

## Abbreviations

ACDF: Anterior cervical decompression and fusion
PF: Posterior foraminotomy
CR: Cervical radiculopathy
CASP: Clinical adjacent segment pathology
RASP: Radiological adjacent segment pathology
ASD: Adjacent segment disease
MCID: Minimum clinically important difference
PROMS: Patient-reported outcome measures
NDI: Neck disability index
NRS: Numeric rating scale
EQ-5D: European Quality of Life-5 Dimensions
CSF: Cerebrospinal fluid

## References

[1] Goldstein B (2002). Anatomic issues related to cervical and lumbosacral radiculopathy. Phys Med Rehabil Clin N Am.

[2] Radhakrishnan K, Litchy WJ, O'Fallon WM, Kurland LT (1994). Epidemiology of cervical radiculopathy. A population-based study from Rochester, Minnesota, 1976 through 1990. Brain..

[3] Panych LP, Madore B (2018). The physics of MRI safety. J Magn Reson Imaging.

[4] Albert TJ, Murrell SE (1999). Surgical management of cervical radiculopathy. J Am Acad Orthop Surg.

[5] Hershman SH, Kunkle WA, Kelly MP, Buchowski JM, Ray WZ, Bumpass DB, et al. Esophageal perforation following anterior cervical spine surgery: case report and review of the literature. Global Spine J. 2017;7(1 Suppl):28S-36S, 10.1177/2192568216687535.Hershman SH, Kunkle WA, Kelly MP, Buchowski JM, Ray WZ, Bumpass DBet al. Esophageal perforation following anterior cervical spine surgery: case report and review of the literature. Global Spine J. 2017;7(1 Suppl):28S-36S, DOI: 10.1177/2192568216687535. 10.1177/2192568216687535 PMC5400185 28451488

[6] Ghobrial GM, Harrop JS, Sasso RC, Tannoury CA, Tannoury T, Smith ZA, et al. Anterior cervical infection: presentation and incidence of an uncommon postoperative complication. Global Spine J. 2017;7(1 Suppl):12S–6S. 10.1177/2192568216687535. 10.1177/2192568216687546 PMC5400186 28451485

[7] Gokaslan ZL, Bydon M, De la Garza-Ramos R, Smith ZA, Hsu WK, Qureshi SA (2017). Recurrent laryngeal nerve palsy after cervical spine surgery: a multicenter AOSpine clinical research network study. Global Spine J..

[8] Tempel ZJ, Smith JS, Shaffrey C, Arnold PM, Fehlings MG, Mroz TE, Riew KD, Kanter AS (2017). A multicenter review of superior laryngeal nerve injury following anterior cervical spine surgery. Global Spine J..

[9] Ames CP, Clark AJ, Kanter AS, Arnold PM, Fehlings MG, Mroz TE, et al. Hypoglossal nerve palsy after cervical spine surgery. Global Spine J. 2017;7(1 Suppl):37S–9S. 10.1177/2192568216687307. 10.1177/2192568216687307 PMC5400183 28451489

[10] Hsu WK, Kannan A, Mai HT, Fehlings MG, Smith ZA, Traynelis VC, et al. Epidemiology and outcomes of vertebral artery injury in 16 582 cervical spine surgery patients: an AOSpine North America multicenter study. Global Spine J. 2017;7(1 Suppl):21S–7S. 10.1177/2192568216686753. 10.1177/2192568216686753 PMC5400180 28451487

[11] O'Neill KR, Fehlings MG, Mroz TE, Smith ZA, Hsu WK, Kanter AS (2017). A multicenter study of the presentation, treatment, and outcomes of cervical dural tears. Global Spine J.

[12] Daniels AH, Hart RA, Hilibrand AS, Fish DE, Wang JC, Lord EL, et al. Iatrogenic spinal cord injury resulting from cervical spine surgery. Global Spine J. 2017;7(1 Suppl):84S–90S. 10.1177/2192568216688188. 10.1177/2192568216688188 PMC5400194 28451499

[13] Traynelis VC, Malone HR, Smith ZA, Hsu WK, Kanter AS, Qureshi SA, et al. Rare complications of cervical spine surgery: Horner’s syndrome. Global Spine J. 2017;7(1 Suppl):103S–8S. 10.1177/2192568216688184. 10.1177/2192568216688184 PMC5400192 28451480

[14] Than KD, Mummaneni PV, Smith ZA, Hsu WK, Arnold PM, Fehlings MG, et al. Brachial plexopathy after cervical spine surgery. Global Spine J. 2017;7(1 Suppl):17S–20S. 10.1177/2192568216687297. 10.1177/2192568216687297 PMC5400182 28451486

[15] Thompson SE, Smith ZA, Hsu WK, Nassr A, Mroz TE, Fish DE, et al. C5 palsy after cervical spine surgery: a multicenter retrospective review of 59 cases. Global Spine J. 2017;7(1 Suppl):64S–70S. 10.1177/2192568216688189. 10.1177/2192568216688189 PMC5400195 28451494

[16] Derakhshan A, Lubelski D, Steinmetz MP, Corriveau M, Lee S, Pace JR, Smith GA, Gokaslan Z, Bydon M, Arnold PM, Fehlings MG, Riew KD, Mroz TE (2017). Thoracic duct injury following cervical spine surgery: a multicenter retrospective review. Global Spine J..

[17] Skeppholm M, Olerud C (2013). Comparison of dysphagia between cervical artificial disc replacement and fusion: data from a randomized controlled study with two years of follow-up. Spine (Phila Pa 1976).

[18] Anderson PA, Andersson GBJ, Arnold PM (2012). al. e. Terminology. Spine..

[19] Hilibrand AS, Robbins M (2004). Adjacent segment degeneration and adjacent segment disease: the consequences of spinal fusion?. Spine J.

[20] Kozak JA, Hanson GW, Rose JR, Trettin DM, Tullos HS (1989). Anterior discectomy, microscopic decompression, and fusion: a treatment for cervical spondylotic radiculopathy. J Spinal Disord.

[21] Brigham CD, Tsahakis PJ (1995). Anterior cervical foraminotomy and fusion. Surgical technique and results. Spine (Phila Pa 1976).

[22] Jagannathan J, Sherman JH, Szabo T, Shaffrey CI, Jane JA (2009). The posterior cervical foraminotomy in the treatment of cervical disc/osteophyte disease: a single-surgeon experience with a minimum of 5 years’ clinical and radiographic follow-up. J Neurosurg Spine..

[23] Ruetten S, Komp M, Merk H, Godolias G (2008). Full-endoscopic cervical posterior foraminotomy for the operation of lateral disc herniations using 5.9-mm endoscopes: a prospective, randomized, controlled study. Spine (Phila Pa 1976).

[24] Lawton CD, Smith ZA, Lam SK, Habib A, Wong RH, Fessler RG (2014). Clinical outcomes of microendoscopic foraminotomy and decompression in the cervical spine. World Neurosurg..

[25] Skovrlj B, Gologorsky Y, Haque R, Fessler RG, Qureshi SA (2014). Complications, outcomes, and need for fusion after minimally invasive posterior cervical foraminotomy and microdiscectomy. Spine J.

[26] Kwon YJ (2014). Long-term clinical and radiologic outcomes of minimally invasive posterior cervical foraminotomy. J Korean Neurosurg Soc.

[27] Branch BC, Hilton DL, Watts C (2015). Minimally invasive tubular access for posterior cervical foraminotomy. Surg Neurol Int.

[28] Oertel JM, Philipps M, Burkhardt BW (2016). Endoscopic posterior cervical foraminotomy as a treatment for osseous foraminal stenosis. World Neurosurg.

[29] Burkhardt BW, Muller S, Oertel JM (2016). Influence of prior cervical surgery on surgical outcome of endoscopic posterior cervical foraminotomy for osseous foraminal stenosis. World Neurosurg..

[30] Peto I, Scheiwe C, Kogias E, Hubbe U (2017). Minimally invasive posterior cervical foraminotomy: Freiburg experience with 34 patients. Clin Spine Surg.

[31] Dunn C, Moore J, Sahai N, Issa K, Faloon M, Sinha K, et al. Minimally invasive posterior cervical foraminotomy with tubes to prevent undesired fusion: a long-term follow-up study. J Neurosurg Spine. 2018;29(4):358–64. 10.3171/2018.2.SPINE171003. 10.3171/2018.2.SPINE171003 29957145

[32] Kim SJ, Seo JS, Lee SH, Bae J (2019). Comparison of anterior cervical foraminotomy and posterior cervical foraminotomy for treating single level unilateral cervical radiculopathy. Spine (Phila Pa 1976).

[33] Clarke MJ, Ecker RD, Krauss WE, McClelland RL, Dekutoski MB (2007). Same-segment and adjacent-segment disease following posterior cervical foraminotomy. J Neurosurg Spine..

[34] MacDowall A, Heary RF, Holy M, Lindhagen L, Olerud C. Posterior foraminotomy versus anterior decompression and fusion in patients with cervical degenerative disc disease with radiculopathy: up to 5 years of outcome from the national Swedish spine register. J Neurosurg Spine. 2019:1–9. 10.3171/2019.9.SPINE19787 31731263

[35] Kumar GR, Maurice-Williams RS, Bradford R (1998). Cervical foraminotomy: an effective treatment for cervical spondylotic radiculopathy. Br J Neurosurg.

[36] Grieve JP, Kitchen ND, Moore AJ, Marsh HT (2000). Results of posterior cervical foraminotomy for treatment of cervical spondylitic radiculopathy. Br J Neurosurg.

[37] Schoggl A, Reddy M, Saringer W, Ungersbock K (2002). Social and economic outcome after posterior microforaminotomy for cervical spondylotic radiculopathy. Wien Klin Wochenschr.

[38] Spurling RG, Segerberg LH (1953). Lateral intervertebral disk lesions in the lower cervical region. J Am Med Assoc.

[39] Frykholm R (1947). Deformities of dural pouches and strictures of dural sheaths in the cervical region producing nerve-root compression; a contribution to the etiology and operative treatment of brachial neuralgia. J Neurosurg.

[40] Thome C, Leheta O, Krauss JK, Zevgaridis D (2006). A prospective randomized comparison of rectangular titanium cage fusion and iliac crest autograft fusion in patients undergoing anterior cervical discectomy. J Neurosurg Spine..

[41] Smith GW, Robinson RA (1958). The treatment of certain cervical-spine disorders by anterior removal of the intervertebral disc and interbody fusion. J Bone Joint Surg Am.

[42] Vernon H (2008). The neck disability index: state-of-the-art, 1991-2008. J Manip Physiol Ther.

[43] Burstrom K, Johannesson M, Diderichsen F (2001). Swedish population health-related quality of life results using the EQ-5D. Qual Life Res.

[44] Strömqvist B, Jönsson B, Froitzell P, Hägg O, Larsson B-E, Lind B (2001). The Swedish national register for lumbar spine surgery: Swedish society for spinal surgery. Acta Orthop Scand.

[45] Parai C, Hagg O, Lind B, Brisby H (2020). ISSLS prize in clinical science 2020: the reliability and interpretability of score change in lumbar spine research. Eur Spine J.

[46] Chan AW, Tetzlaff JM, Gotzsche PC, Altman DG, Mann H, Berlin JA, et al. SPIRIT 2013 explanation and elaboration: guidance for protocols of clinical trials. BMJ. 2013;346(jan08 15):e7586. 10.1136/bmj.e7586. 10.1136/bmj.e7586 PMC3541470 23303884

[47] Boutron I, Altman DG, Moher D, Schulz KF, Ravaud P, Group CN (2017). CONSORT statement for randomized trials of nonpharmacologic treatments: a 2017 update and a CONSORT extension for nonpharmacologic trial abstracts. Ann Intern Med.

[48] Carreon LY, Glassman SD, Campbell MJ, Anderson PA (2010). Neck disability index, short form-36 physical component summary, and pain scales for neck and arm pain: the minimum clinically important difference and substantial clinical benefit after cervical spine fusion. Spine J.

[49] Young IA, Cleland JA, Michener LA, Brown C (2010). Reliability, construct validity, and responsiveness of the neck disability index, patient-specific functional scale, and numeric pain rating scale in patients with cervical radiculopathy. Am J Phys Med Rehabil.

[50] Parker SL, Godil SS, Shau DN, Mendenhall SK, McGirt MJ (2013). Assessment of the minimum clinically important difference in pain, disability, and quality of life after anterior cervical discectomy and fusion: clinical article. J Neurosurg Spine.

[51] White AA, Johnson RM, Panjabi MM (1975). Southwick WO Biomechanical analysis of clinical stability in the cervical spine. Clin Orthop Relat Res.

[52] Stromqvist B, Fritzell P, Hagg O, Jonsson B, Swedish Society of Spinal S. The Swedish spine register: development, design and utility, Eur spine J. 2009;18(Suppl 3):294–304. 10.1007/s00586-009-1043-4 PMC2899320 19495812

[53] Tyson JE, Pedroza C, Wallace D, D'Angio C, Bell EF, Das A (2016). Stopping guidelines for an effectiveness trial: what should the protocol specify?. Trials..

[54] White IR, Royston P, Wood AM (2011). Multiple imputation using chained equations: issues and guidance for practice. Stat Med.

[55] Sahai N, Changoor S, Dunn CJ, Sinha K, Hwang KS, Faloon M, et al. Minimally invasive posterior cervical foraminotomy as an alternative to anterior cervical discectomy and fusion for unilateral cervical radiculopathy: a systematic review and meta-analysis. Spine (Phila Pa 1976). 2019. 10.1097/BRS.0000000000003156 31343619

[56] MacDowall A, Canto Moreira N, Marques C, Skeppholm M, Lindhagen L, Robinson Y, et al. Artificial disc replacement versus fusion in patients with cervical degenerative disc disease and radiculopathy: a randomized controlled trial with 5-year outcomes. J Neurosurg Spine. 2019;30(3):323–31. 10.3171/2018.9.SPINE18659. 10.3171/2018.9.SPINE18659 30641852

[57] Delamarter RB, Zigler J (2013). Five-year reoperation rates, cervical total disc replacement versus fusion, results of a prospective randomized clinical trial. Spine (Phila Pa 1976).

