# Correction to: Operative treatment of cervical radiculopathy: anterior cervical decompression and fusion compared with posterior foraminotomy: study protocol for a randomized controlled trial

**DOI:** 10.1186/s13063-021-05623-9

**Published:** 2021-09-29

**Authors:** Marek Holy, Anna MacDowall, Freyr Gauti Sigmundsson, Claes Olerud

**Affiliations:** 1 Department of Orthopedic Surgery, Örebro University School of Medical Sciences, Örebro University Hospital, Örebro, Sweden; 2 Department of Surgical Sciences, Uppsala University Hospital, Uppsala, Sweden

**Correction to: Trials 22, 607 (2021)**

**https://doi.org/10.1186/s13063-021-05492-2**

Following the publication of the original article [1], we were notified of a typo in the name of the 4th author.

- Originally published name: Class Olerud
- Correct name: Claes Olerud

The original article has been corrected.
